# 1,3,4-Oxadiazole Derivatives of Pyrrolo[3,4-*d*]pyridazinone Alleviate TNBS-Induced Colitis and Exhibit No Significant Testicular Toxicity

**DOI:** 10.3390/ph18040546

**Published:** 2025-04-08

**Authors:** Anna Merwid-Ląd, Piotr Ziółkowski, Beata Nowak, Piotr Świątek, Łukasz Szczukowski, Joanna Kwiatkowska, Katarzyna Piasecka, Adam Szeląg, Marta Szandruk-Bender

**Affiliations:** 1 Department of Pharmacology, Wroclaw Medical University, Mikulicza-Radeckiego 2, 50-345 Wrocław, Poland; beata.nowak@umw.edu.pl (B.N.); adam.szelag@umw.edu.pl (A.S.); marta.szandruk@umw.edu.pl (M.S.-B.); 2 Department of Clinical and Experimental Pathology, Wroclaw Medical University, Marcinkowskiego 1, 50-368 Wrocław, Poland; piotr.ziolkowski@umw.edu.pl; 3 Department of Medicinal Chemistry, Wroclaw Medical University, Borowska 211, 50-556 Wrocław, Poland; piotr.swiatek@umw.edu.pl (P.Ś.); lukasz.szczukowski@umw.edu.pl (Ł.S.)

**Keywords:** testicular toxicity, experimental colitis, 1,3,4-oxadiazole, pyrrolo[3,4-*d*]pyridazinone derivatives, MDA, SOD, LDH, IL-1, MMP-9, histology

## Abstract

**Background/Objectives:** Inflammatory bowel disease significantly impairs the patient’s quality of life. In young individuals, both the disease and the drugs used for the treatment may impact fertility. Our study aimed to assess the action of new 1,3,4-oxadiazole derivatives of pyrrolo[3,4-d]pyridazinone on the rat testes in a model of TNBS-induced colitis in rats. **Methods:** In the current study, testes from eight randomly chosen rats were taken from each of the following groups: the control group (K), the colitis group (C), and the groups receiving compounds 7b, 10b, and 13b in higher doses (20 mg/kg). **Results:** Colitis did not affect the testicular index (expressed as a percentage of the body weight), but in group 13b, this parameter was significantly higher than in group K. No significant differences between groups were noticed in malondialdehyde, superoxide dismutase, interleukin-1, or metalloproteinase 9 levels. In the colitis group, lactate dehydrogenase activity in the testes was not increased; however, the administration of compound 10b significantly increased this parameter when compared to both groups K and C. Histological evaluation also did not reveal abnormalities, and the morphology of the testicular tissues was comparable in all groups. **Conclusions:** The results may suggest that the new 1,3,4-oxadiazole derivatives of pyrrolo[3,4-*d*]pyridazinone did not exert significant testicular toxicity.

## 1. Introduction

Despite some growing trends for not having children [1], many couples would like to have offspring, and infertility is a concern for them. It is estimated that 8–12% of couples may experience problems with conception [2]. According to other sources, the percentage may be even higher, reaching up to 15–20% [3]. The male factor as a sole reason for infertility may vary depending on the country and is estimated for about 20–30% of cases, but may be as high as 50% [4,5]. Data provided by infertility centers in Poland estimate that the male factor in infertility accounts for as much as 55% [6,7]. The problem of declining sperm quality is disputable, but some available data suggest a decline in sperm counts over the years [8,9]. It is not well confirmed whether observed changes in sperm count also impact fertility [10]. Some studies suggest that in patients with inflammatory bowel disease (IBD), especially ulcerative colitis type, sperm quality and testosterone levels decrease along with the greater severity of the disease [11,12,13], and normalization of testosterone levels by its supplementation improves the activity of the IBD [11].

Among many reasons for infertility, certain drugs and diseases may be important. IBD is a tremendous health problem worldwide. Its incidence has increased over the past decades, especially in developing countries, in areas usually considered to have a low prevalence of this disease. Environmental factors are often considered an essential risk for IBD development [12]. The global changes in lifestyle and diet, along with individual genetic factors, immunological state, and specific microbiota, influence the occurrence of the disease. Although IBD mainly affects the gastrointestinal tract, as a chronic inflammatory state, it may also affect other tissues and organs and impair male reproduction [13]. IBD affects all age groups of patients, including children, young adults planning parenthood, and older people [14,15], with the peak onset occurring between 15 and 30 years of age [16]. It is not well established if IBD directly influences male fertility because some patients decide not to conceive a child during the active period of the disease [17]. Underlying IBD is the reason for the lack of sexual activity in about half of the male patients who are sexually inactive [18], and it may be a multifactorial problem, including low self-esteem and concomitant depression [16]. It is estimated that the rate of sexual dysfunction in men with IBD is three to five times higher than in the general population (15–25% vs. 5%, respectively), which not only significantly impairs the quality of life of these patients but also requires a variety of treatment modalities at a young age [15]. The study of Wdowiak et al. [19] revealed that worse semen parameters in men with ulcerative colitis might also be found when the patients are in remission. The severity of the disease negatively correlates with sperm parameters such as sperm concentration or rapid progressive motility, but positively correlates with parameters such as immotile sperm or morphological abnormalities. Testicular injury may also be the consequence of many other generalized inflammatory conditions, such as rheumatoid arthritis [20] or systemic lupus erythematosus [21], metabolic diseases, e.g., diabetes mellitus [22,23], obesity [24], chronic hypoxia [25], or environmental pollution [26].

Testes are a crucial element of the male reproductive system. The specific blood–testicular barrier (BTB) was developed to protect spermatogenesis from external factors and toxins. One of the essential components of the testicular barrier is the network of Sertoli cells, which creates a physical part of it. In the basal membrane of Sertoli cells, peritubular myoid cells, and Leydig cells, a variety of transporters are located that form the physiological part of the barrier [27]. Next, this highly specialized barrier also acts as an immunological barrier and creates a specific environment for the development of germ cells [27,28,29]. In physiological conditions, most drugs do not easily permeate through the BTB because of efflux pumps that transport drugs back into circulation. Moreover, different factors, including chemical substances or diseases, may disrupt the integrity of the testicular barrier [27,30,31], leading to greater permeation of xenobiotics with subsequent testicular injury and possible problems with fertility.

Many agents may exert toxic effects on the male reproductive system, including both testicular tissue and epididymides [32]. The impact of antiandrogens is rather apparent, but the mechanisms of the reproductive toxicity of other drugs are not always established. It has been known for many years that some conventional medications used for IBD, such as sulfasalazine, methotrexate, or azathioprine, may cause transient oligospermia, increase the number of abnormal forms, impair semen motility, or even cause morphological changes in testes [32,33,34,35,36]. In animal in vivo studies, sulfasalazine was found to worsen semen parameters and alter testicular morphology, along with increased markers of oxidative stress and decreased plasma testosterone levels [37]. The active metabolite of sulfasalazine, sulfapyridine, was found to induce oxidative stress that may be responsible for reproductive toxicity [33]. Next to the pathologies in sperm count and motility, sulfasalazine was found to increase the relative weight of epididymis and seminal vesicles with alterations of oxidative stress parameters—increased malondialdehyde and decreased antioxidants in both testes and epididymis of rats [38]. However, those actions seem to be reversible after discontinuing the treatment. Substituting sulfasalazine, e.g., for balsalazide, may provide some benefits [39]. The action of mesalamine on the reproductive system is less pronounced but may also cause reversible oligospermia in individual cases. However, patients who switched from sulfasalazine to 5-aminosalicylic acid revealed improved fertility [32,34,40,41]; nonetheless, data concerning the benefits of switching sulfasalazine to mesalamine are more controversial [39]. In the case of many immunosuppressant agents, changes in sperm parameters are reversible during treatment or upon its termination. Still, effective contraception is recommended, as well as suggestions for cryopreservation of semen before the onset of therapy [32]. In the case of the newer biological agents used in IBD or rheumatological disease, data concerning their testicular toxicity are scant and controversial. On the one hand, it was shown that infliximab might protect from cadmium-induced testicular toxicity without exerting toxicity on testes morphology, iNOS expression, or oxidative stress parameters [42], but on the other hand, infliximab might block the TNF-alpha-induced survival of cells in the rat seminiferous epithelium [43]. Some concerns about testicular toxicity were also raised from the preclinical studies of the Janus kinase inhibitor filgotinib [44].

We have previously studied new derivatives of 1,3,4-derivatives of pyrrolo[3,4-*d*]pyridazinone, inhibiting cyclooxygenase activity in various models of pain and inflammation [45,46]. Their efficacy in a rat model of 2,4,6-trinitrobenzenesulfonic acid (TNBS)-induced colitis was also evaluated [47]. We demonstrated that these compounds normalize the level of a variety of proinflammatory mediators and reduce the influx of inflammatory cells to the site of the ongoing inflammation [45,46,47]. Since cyclooxygenase 2 (COX-2) and prostaglandin E2 play an important role in regulating the Th17/Treg axis (involved in the pathogenesis of IBD), we examined whether these compounds affect the Th17/Treg axis. In the TNBS colitis, compounds 7b and 13b targeted the Th17 developmental pathway by reducing the expression of Th17-specific transcription factors and the Th17 cell-polarizing cytokine. Compound 10b targeted the Treg developmental pathway by enhancing the expression of a Treg-specific transcription factor [47]. Promising results of analgesic and anti-inflammatory actions, as well as an improvement in chemically induced colitis with substances marked as 7b, 10b, and 13b, which have significantly lower toxicity to the gastric mucosa than indomethacin, raised the question of the toxicity of these compounds to distinct organs, including testes. The impact of those compounds on, e.g., oxidative stress parameters or the morphological structure of testes was not evaluated earlier. This may be interesting in the context of potential long-term use of such substances, not only in IBD treatment but as analgesic or anti-inflammatory agents. In this context, assessing the reproductive toxicity of compounds with potential activity in IBD seems very important.

Following the 3R (replacement, reduction, and refinement) principles, the biobanked testicular tissue samples were used for the current study, which aimed to assess the influence of 1,3,4- derivatives of pyrrolo[3,4-*d*]pyridazinone (compounds 7b, 10b, and 13b) on selected injury, oxidative stress, and inflammatory parameters in rat testes. The results may give insight into the safety of those new substances with important analgesic and anti-inflammatory properties.

## 2. Results

### 2.1. Body Weight and Testes Index

**Figure 1:**
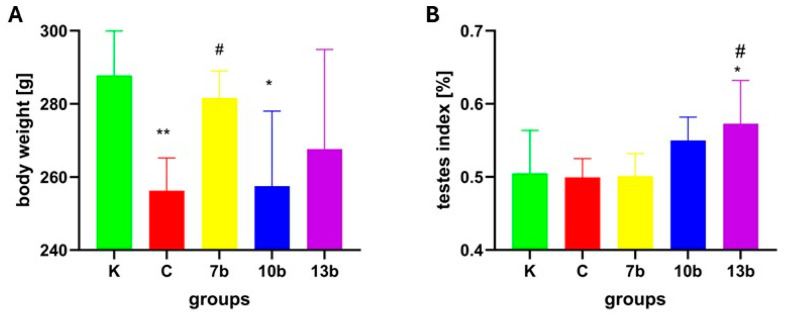
Body weight changes (**A**) and testes index (**B**) in the studied groups (N = 8); K—control group—receiving 0.5% of carboxymethylcellulose (CMC) solution intragastrically (*i.g.*) and normal saline solution rectally, C—colitis group—receiving 0.5% CMC *i.g.* and solution of 2,4,6-trinitrobenzenesulfonic acid (TNBS) rectally, 7b, 10b, and 13b—groups receiving *i.g.* studied substances at 20 mg/kg doses and rectally TNBS solution. * reflects differences to the control group (K), *p* < 0.05. ** reflects differences to the control group (K), *p* < 0.01. ^#^ reflects differences to the colitis group (C), *p* < 0.05.

On the last day of the experiment, the body weight of animals randomly selected for this part of the study was the lowest in the colitis group (C) and significantly lower than in the control group (K) (group C vs. K, *p* < 0.01). Additionally, a significant decrease was observed in the group receiving compound 10b (group 10b vs. K, *p* < 0.05). Only administration of compound 7b prevented the decrease in body weight (group 7b vs. C, *p* < 0.05 and 7b vs. K, *p* = NS) (Figure 1A)

Colitis did not change the testis index. The highest testis indexes were noticed in groups receiving compounds 10b and 13b, but only in the case of group 13b was the difference significant when compared to the colitis group (13b vs. C, *p* < 0.05). The testis index in group 13b was also significantly higher than in the control group (13b vs. K, *p* < 0.05) (Figure 1B).

### 2.2. Oxidative Stress Parameters

**Figure 2:**
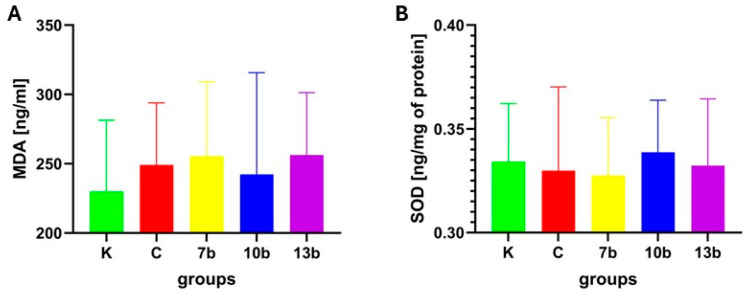
Malondialdehyde (MDA) concentration (**A**) and superoxide dismutase (SOD) activity (**B**) in testicular homogenates in the studied groups (N = 8). K—control group—receiving 0.5% of carboxymethylcellulose (CMC) solution intragastrically (*i.g.*) and normal saline solution rectally, C—colitis group—receiving 0.5% CMC *i.g.* and solution of 2,4,6-trinitrobenzenesulfonic acid (TNBS) rectally, 7b, 10b, and 13b—groups receiving *i.g.* studied substances at 20 mg/kg doses and rectally TNBS solution. No significant differences were noticed between the studied groups.

Despite the increase in malondialdehyde (MDA) concentration in the testis tissue in all groups (colitis and all groups receiving studied compounds), no significant differences were noticed between the groups (Figure 2A).

Similarly, no significant differences were found between groups in the activity of superoxide dismutase (SOD) (Figure 2B).

### 2.3. Tissue Injury and Inflammatory Parameters

**Figure 3:**
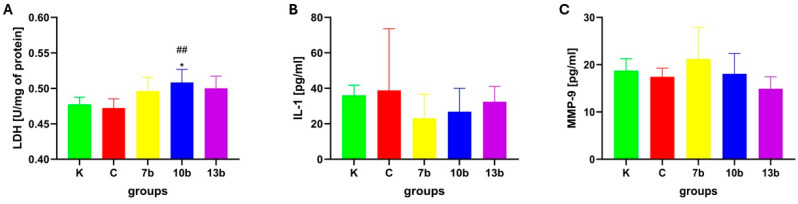
Lactate dehydrogenase (LDH) activity (**A**), interleukin-1β (IL-1β) (**B**), and matrix metalloproteinase 9 (MMP-9) (**C**) concentrations in the testis tissue homogenates (N = 8). K—control group—receiving 0.5% of carboxymethylcellulose (CMC) solution intragastrically (*i.g.*) and normal saline solution rectally, C—colitis group—receiving 0.5% CMC *i.g.* and solution of 2,4,6-trinitrobenzenesulfonic acid (TNBS) rectally, 7b, 10b, and 13b—groups receiving *i.g.* studied substances at 20 mg/kg doses and rectally TNBS solution. * reflects differences to the control group (K), *p* < 0.05. ^##^ reflects differences to the colitis group (**C**), *p* < 0.01.

From all the groups studied, the highest activity of lactate dehydrogenase was detected in the group receiving compound 10b, and the value was significantly higher than in the control group (10b vs. K, *p* < 0.05) and the colitis group (10b vs. C, *p* = 0.01). Colitis did not affect the LDH activity in the testicular tissue (Figure 3A).

The concentrations of interleukin-1 were not significantly different in all studied groups, with the lowest value found in the group receiving the compound 7b.

The activities of matrix metalloproteinase 9 were comparable in all studied groups. No significant differences were observed.

### 2.4. Histological Evaluation

**Figure 4:**
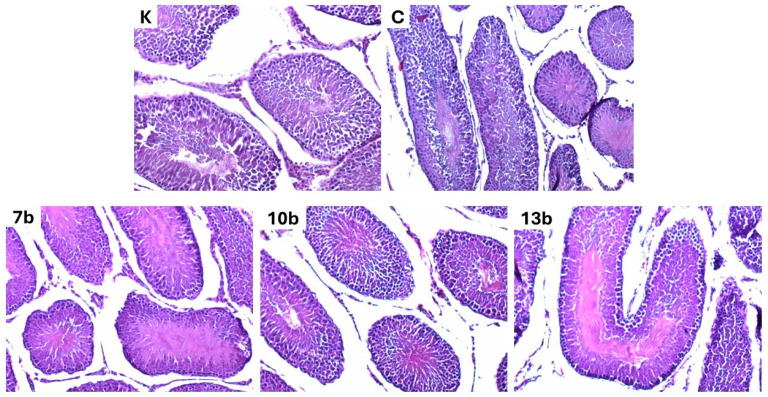
Histological evaluation of testicular tissue in control (K), colitis (C), and groups 7b, 10b, and 13b receiving studied compounds at doses of 20 mg/kg (N = 8). K—control group—receiving 0.5% of carboxymethylcellulose (CMC) solution intragastrically (*i.g.*) and normal saline solution rectally, C—colitis group—receiving 0.5% CMC *i.g.* and solution of 2,4,6-trinitrobenzenesulfonic acid (TNBS) rectally, 7b, 10b, and 13b—groups receiving *i.g.* studied substances at 20 mg/kg doses and rectally TNBS solution.

The morphology of the testicular tissue was not affected in all studied groups. Neither colitis nor the administered substances caused abnormalities. Apart from minor signs of hyperemia, the histological picture does not differ from the norm. Representative images of testes samples from each of the studied groups are presented in Figure 4.

Eosin-hematoxylin staining; all images were taken at 100×magnification. Histopathological examination of tissues in the control group revealed a normal histological pattern of the testis. This comprised seminiferous tubules lined by layers of germinal epithelium and Sertoli cells, separated by interstitial Leydig cells. Spermatogenic cells showed different stages of maturation. No significant changes in testicular morphology in the studied groups were noticed compared to the control.

## 3. Discussion

In the in silico and in vitro studies of Szczukowski et al. [48], it was found that all three studied compounds—7b, 10b, and 13b—have shown a better affinity for the cyclooxygenase 2 (COX-2) isoenzyme than for cyclooxygenase 1 (COX-1) and a stronger COX-2 inhibitory effect than the reference drug, meloxicam. The 13b compound may be considered a selective COX-2 inhibitor, while the compound 7b seems to be the most active in inhibiting COX-1 activity. Compounds 10b and 13b were also checked for their antinociceptive activity. Both substances exerted dose-dependent action in noxious stimuli-induced models of pain (the tail-flick test and the formalin test) and counteracted inflammatory nociception (based on the second phase of the formalin test results). The anti-inflammatory properties of compounds 10b and 13b were further confirmed in the carrageenan test, reducing paw edema [45,46]. Both substances revealed no significant gastric toxicity, which may result from replacing the free carboxylic group with a bioisosteric moiety, 1,3,4-oxadiazole-2-thione. The free carboxylic group, which is characteristic of most nonsteroidal anti-inflammatory drugs, may cause mucosal damage by local irritation, and its replacement by 1,3,4-oxadiazole-2-thione results in better affinity to the COX-2 isoform and reduced gastrotoxicity [46]. Based on those presumptions, compounds 7b, 10b, and 13b were used in the TNBS-induced colitis model in rats, in which compounds 7b and 13b exerted significant protective effects on colon tissue [47]. In the cited study, all three compounds were administered for 16 days, which raises the risk of adverse effects or tissue toxicity. Therefore, we decided to use testicular tissue from the biobank to check whether, in addition to the efficacy of studied substances in treating induced colitis in rats, they do not demonstrate toxicity to the testes.

Some early data from animal studies suggested that cyclooxygenase activity and prostaglandins are insignificant for male fertility and testicular function [49]. However, COX-2 was detected in testicular tissue in infertile patients with impaired spermatogenesis. The expression of prostaglandin receptors was found on Sertoli cells, and it is postulated that prostaglandins may exert an inhibitory effect on spermatogenesis [50]. It seems that COX-1 and COX-2 regulate undifferentiated spermatogonia/spermatogonial stem cell differentiation in a different way [51], and constitutive expression of COX-2 was detected in immature and mature rat testes, mainly in the spermatogonia [52]. Localization and activity of the COX-1 and COX-2 enzymes may change during the development, from the prepubertal stage through adulthood to the elderly [53].

Many different nonsteroidal anti-inflammatory drugs (NSAIDs) that are COX inhibitors are used as analgesic, antipyretic, or anti-inflammatory medications for shorter or longer durations. They differ in chemical structure and selectivity to COX isoforms. Many papers describe testicular injury after the administration of diclofenac. Alterations were found, among others, in the reproductive organ relative weight and morphology, oxidative stress parameters, lactate dehydrogenase activity, and semen quality [54,55,56,57,58]. Nevertheless, there are also reports about the toxicity of other NSAIDs. Naproxen and meloxicam impaired sperm count and motility in rats with damage to seminiferous tubules and an imbalance in the oxido–redox state in testicular tissue. This toxicity is considered direct since plasma levels of gonadal hormones remained unchanged [59]. Another drug, nimesulide, administered to prepubertal rats, caused significant differences in testosterone and estradiol levels without substantial changes in testicular architecture [60]. However, experimental studies sometimes give contradictory results on the impact of COX inhibitors on the reproductive system. After a single intragastric administration, indomethacin caused an imbalance between pro- and antioxidants in rat testes [61]. On the other hand, the same drug exerted a protective effect in testicular tissue exposed to noxious stimuli, such as vanadium, increasing steroid hormone synthesis and normalizing the affected parameters of oxidative stress [62]. Data concerning selective COX-2 inhibitors, such as celecoxib, rather suggest their protective action, especially in the case of testicular damage caused by inflammation [63] or herbicides [64]. It is also worth noting that maternal exposure to NSAIDs may pose a risk of pathologies in the reproductive system of male offspring [65], such as a decreased number of Sertoli and Leydig cells [66].

In the current study, we evaluated such parameters as relative testicular weight (testes index), basic parameters of oxido–redox state (MDA level and SOD activity), as well as parameters of tissue injury (LDH activity and MMP-9 concentration), and proinflammatory cytokine—IL-1β level. All parameters were measured in testicular tissue homogenates. In addition to the abovementioned markers of tissue damage, the histological evaluation of testis morphology under standard hematoxylin–eosin staining was performed. To our knowledge, it is the first study evaluating the testicular safety/toxicity of the 1,3,4-derivatives of pyrrolo[3,4-*d*]pyridazinone; therefore, we decided to check the basic parameters of various injury pathways.

In this model of TNBS-induced colitis, we did not notice any impact of the colitis on most of the parameters studied, except for the body weight at the end of the experiment. It contrasts with other colitis models, where the body weight decrease in the colitis group (positive control) was also noticed; however, alterations in testicular morphology, sperm characteristics, oxidative stress parameters, or inflammatory markers in colitis groups were significant. This may be explained, first of all, by the different times between colitis induction and the evaluation of a variety of injury factors or the model used [67,68]. The main experiment was designed to check the preventive action of compounds 7b, 10b, and 13b in the colitis model. The substances were administered before the induction of colitis with TNBS administration. After the induction of colitis, animals were sacrificed after 48 h, which may not be enough time to find the impact of colitis on the parameters we studied in testicular tissue. The second reason for discrepancies may be the colitis model used. In our basal experiment, rectal administration of TNBS to rats was performed. In contrast, in the earlier cited papers of Azmy et al. and Farombi et al. [67,68], oral induction of colitis with dextran sodium sulfate (DSS) was used, which, for sure, may lead to more diffuse pathologies in the gastrointestinal tract with much more pronounced systemic response to the colitis.

We noticed that the substance 13b, administered at a 20 mg/kg dose for 16 days, increased the testis index compared to the control group. It is difficult to explain this based on the currently available data. There was a decrease in epididymal relative weight after sulfasalazine administration [38], but, e.g., administration of nimesulide for 56 days did not significantly change either absolute or relative testis weights [60]. We, however, have found some degree of hyperemia in histological evaluation; however, it was similar in all samples that were blindly assessed.

Oxidative stress is a two-edged sword in cells and tissues, including the male reproductive system. Such processes as capacitation, hyperactivation, acrosomal reaction, and fertilization would not be possible without the involvement of oxidative species. However, the excess of free radicals that the antioxidants cannot scavenge leads to sperm damage and problems with fertility [69]. Whereas the negative impact of non-selective COX inhibitors, such as diclofenac, on oxidative stress parameter balance has been described in many papers, the action of selective COX-2 inhibitors is less clear. Diclofenac in various models of testicular injury increased prooxidants and decreased antioxidant state by significant depletion of superoxide dismutase, catalase, glutathione peroxidase activities, as well as testicular glutathione (GSH) concentration [55,58,68]. Data about the action of selective COX-2 inhibitors on the oxido–redox state in the reproductive system are scant. It was found that meloxicam caused some reduction in MDA levels with the increase in GSH concentration in rat kidneys [70], and celecoxib ameliorated the outcome of neonatal necrotizing enterocolitis in rats by improvement of prooxidants/antioxidants balance [71]. All three compounds used in the study of Szandruk-Bender et al. [47] on TNBS-induced colitis inhibit COX-2 activity. It might be suspected that their impact on the tissue oxidative stress parameters resembles meloxicam or celecoxib action more than diclofenac. Indeed, we did not reveal significant disturbances in the MDA levels or SOD activity in testicular homogenates. Some disquieting trends were noticed in the case of compound 10b, with the highest increase in MDA level and the lowest SOD activity. This requires a much more detailed investigation since, in the current study, only two main oxidative parameters were evaluated. To be able to draw conclusions regarding the pro- or antioxidant activity of these new derivatives, it is necessary to determine a broader panel of oxidative stress parameters in the future.

Lactate dehydrogenase is an enzyme found in almost all tissues and plays a crucial role in the anaerobic metabolic pathway. Its activity, on one hand, is an indicator of physiological tissue remodeling. Still, on the other hand, increased activity is measured in numerous pathological conditions [72] or in testes during some drug administration, e.g., levofloxacin [73] or indomethacin [61]. It is worth mentioning that tissue necrosis leads to increased LDH activity in plasma. The LDHC isoenzyme is specific for testicular tissue, especially sperm, constituting about 80% of LDH activity, and is crucial for energy metabolism during spermatogenesis. LDH may be a staging marker in some cancers of the testes [72,74]. Our experiment showed that LDH activity was significantly higher in the group receiving compound 10b than in the control group. It may suggest that this agent caused some potential injury to the testicular tissue. Since the increased LDH activity may also result from pathological anaerobic conditions or inflammation [72], we cannot exclude these factors as a reason for increased LDH in group 10b. However, it was not confirmed in alterations of other parameters, e.g., increased IL-1β level of altered morphology of testes from this group. Of course, we did not distinguish the isoforms of the enzyme. A more detailed evaluation will be necessary in the future. On the other hand, compound 10b was the least effective in alleviating the colitis symptoms or macro- and microscopic damages in TNBS-induced colitis in the main experiment [47], so it is not highly probable that we will use this compound in further studies.

Matrix metalloproteinase 9 is essential in extracellular breakdown, which is crucial in many tissues for growth and remodeling. However, increased expression and activity of MMP-9 may cause the imbalance between extracellular matrix synthesis and its degradation, inflammation, and, e.g., impaired wound healing, which was found in some diseases [75,76]. Over 20 years ago, Siu et al. [77] demonstrated that MMP-9 expression was induced in Sertoli cells after stimulation with TNF-alpha, which may disturb the inter-Sertoli cell tight junctions and disorganize the BTB. Later, some other studies confirmed that alongside the increased expression of MMP-9, increased BTB permeability and decreased occludin levels were noticed [78]. Impaired tight junctions in Sertoli cells may lead to disorders of spermatogenesis. MMP-9 mRNA expression was confirmed in canine and human testes, epididymis, and semen. Increased MMP-9 levels were found in ejaculate samples with low sperm counts [79]. Matrix metalloproteinases play a significant role in semen liquefaction [79,80,81]. Considering these facts about MMP-9, we decided to check whether the administration of compounds 7b, 10b, and 13b for 16 days in the experimental colitis altered the MMP-9 tissue concentrations. We did not reveal any statistically significant differences between the studied groups, either in comparison to the control and colitis groups or between the groups receiving experimental substances. It may be expected that the administration of our studied compound will not interfere with BTB or semen morphology and function, but it, of course, requires specialized evaluation of semen samples.

Interleukin 1-beta is one of the major proinflammatory cytokines [82], and its increased concentration is found in testes in a variety of systemic or local pathological processes, e.g., obesity [83], ischemia/reperfusion injury [84], or some drugs, such as methotrexate [85]. Apart from that, in the male reproductive system, a bilateral connection between COX-2 and interleukins, including IL-1β, was described. The COX-2 pathway serves as a regulatory factor for IL-1β expression, and IL-1β may induce COX-2 expression in different cells. IL-1β regulates the activity of Sertoli cells, which impacts spermatogenesis, or may impact progenitor Leydig cells [86,87]. Taking into account previous data that our tested substances, mainly 13b and 7b, serve as COX-2 inhibitors [48], we decided to investigate whether TNBS-induced colitis model may generate an inflammatory response in the testes and what impact the studied substances have on the testicular level of IL-1β. As discussed earlier, colitis did not change the IL-1β amount in the testes compared to the control group. Studied substances insignificantly decreased the IL-1β levels, with the most visible action of compound 7b (decrease by 36% compared to the control group). Although we expected a more pronounced reduction in IL-1β in the testes, it cannot be excluded that the presence of an inflammatory process may be necessary to reveal the full effect of these compounds.

In animal models, histological alterations in testicular tissue after the administration of NSAIDs are pretty well documented, especially in the case of diclofenac. Most commonly, the degeneration of seminiferous tubules, desquamation of the spermatogenic epithelium, and interstitial edema were noticed [54,55,56,58]. Vyas et al. showed that the severity of histological pathologies was dose-dependent [57]. Less information is available for newer, more selective COX-2 inhibitors. Long-term administration of nimesulide was connected only to some mild degenerative changes in testis morphology [60], and the 4-week celecoxib regimen did not alter the testis tissue histology [88]. Testicular tissue evaluated histologically in our study revealed only a slight level of hyperemia in all examined samples. It may rather exclude the impact of colitis or toxicity of given substances but suggest the influence of factors that were common to all animals. Since the same procedures were used during euthanasia, we suspect that there was a reason for testis hyperemia. We may only speculate that, e.g., hypoxia during the animals’ death was one of the possible reasons since it was demonstrated that hypoxia conditions caused by decreased oxygen supply lead to increased blood flow in testes to sustain proper oxygenation [89].

We are aware that our study has some limitations, but in this preliminary assessment, we would like to follow the 3R principles and reduce the number of animals used for the study by using the tissues that were biobanked. Therefore, we did not plan a separate toxicity experiment. Firstly, using the testicular tissue from the biobank, we could not check the epididymal tissue, which was not biobanked, or evaluate the sperm parameters, which should have been performed immediately after the animals were sacrificed. Secondly, the main study did not assess the impact of substances 7b, 10b, and 13b without the induction of colitis. However, in the current study, we did not notice the impact of colitis on the evaluated parameters. Thirdly, we did not have the possibility to evaluate the expression of some parameters, such as caspase-3, using immunohistochemistry.

## 4. Materials and Methods

### 4.1. General Organization of the Study

**Figure 5:**
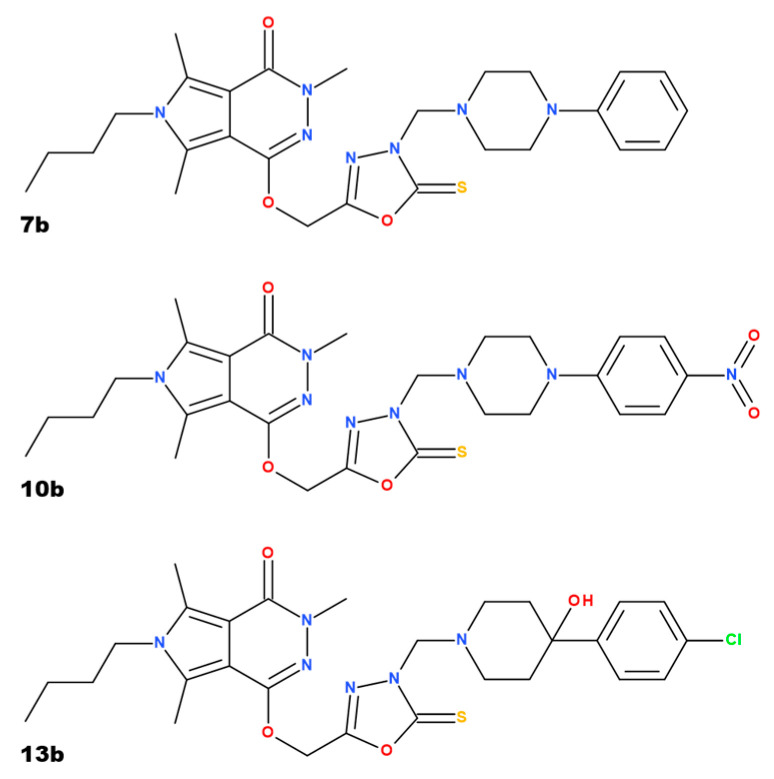
The structures of the investigated derivatives of pyrrolo[3,4-*d*]pyridazinone 7a, 10b, and 13b and their chemical names. Compound 7b—6-butyl-3,5,7-trimethyl-1-[[3-[(4-phenylpiperazin-1-yl)methyl]-2-thioxo-1,3,4-oxadiazol-5-yl]methoxy]pyrrolo[3,4-*d*]pyridazin-4-one; compound 10b—6-butyl-3,5,7-trimethyl-1-[[3-[[4-(4-nitrophenyl)piperazin-1-yl]methyl]-2-thioxo-1,3,4-oxadiazol-2-yl]methoxy]pyrrolo [3,4-*d*]pyridazin-4-one, and compound 13b—6-butyl-1-[[3-[[4-(4-chlorophenyl)-4-hydroxy-1-piperidyl]methyl]-2-thioxo-1,3,4-oxadiazol-5-yl]methoxy]-3,5,7-trimethyl-pyrrolo [3,4-*d*]pyridazin-4-one [48]. Created in BioRender. Nowak, B. (2025) https://BioRender.com/z6gkax3.

The study was performed on the biobanked testis tissue obtained from the earlier study conducted under the approval of the Local Ethics Committee for Animal Experiments in Wrocław at Hirszfeld Institute of Immunology and Experimental Therapy of the Polish Academy of Sciences (005/2020, 15.01.2020). The study assessed the influence of 1,3,4-derivatives of pyrrolo [3,4-*d*]pyridazinone, named 7b, 10b, and 13b, on the course of TNBS-induced experimental colitis in rats. Different spectroscopic and analytical techniques confirmed the structure and purity (>95%) of all compounds based on NMR and ESI-MS spectra. The characteristics of the studied compounds (7b, 10b, and 13b), synthesis pathways, and their chemical structures are described in the paper of Szczukowski et al. [48]. Some data suggested that replacing the free carboxyl group of classic NSAIDs with a bioisosteric group of similar size and lower acidity, e.g., a five-membered heterocyclic ring of 1,3,4-oxadiazole, allows for a meaningful reduction of gastrotoxicity with a simultaneous increase in affinity for COX-2. Moreover, these five-membered heterocycles are essential structural elements of many molecules with significant anti-inflammatory and analgesic activities [90,91,92]. The goal of the synthesis of 1,3,4-oxadiazole derivatives of pyrrolo [3,4-*d*]pyridazinone was to create a new class of effective COX inhibitors, which would have a significant affinity for the inducible COX-2 isoenzyme, while being safe and having no harmful impact on the digestive system [47,48,93]. The structure and chemical names of the compounds are presented in Figure 5.

The detailed experiment design was described in the publication of Szandruk-Bender et al. [47]. Shortly, male Wistar rats were given intragastrically (*i.g.*) three substances, 7b, 10b, and 13b, for 16 consecutive days at doses of 10 mg/kg and 20 mg/kg suspended in 0.5% carboxymethylcellulose (CMC) in a volume of 4 mL/kg; the control group (K) and colitis group (C) received only 0.5% CMC *i.g.* at the same volume. On day 15th, colitis in groups C, 7b, 10b, and 13b was induced by rectal administration of an ethanolic solution of TNBS (50 mg/kg) under isoflurane anesthesia. Rats in the control group K received a normal saline solution rectally in the same volume. All animals were euthanized 48 h after the induction of colitis. Blood and tissue samples were collected for further assessment.

In the current study, testes from 8 randomly chosen rats were taken from each of the following groups: the control group (K), colitis group (C), and groups receiving compounds 7b, 10b, and 13b in higher doses (20 mg/kg). Corresponding samples (from the same rats) in formalin were prepared for histological evaluation, and frozen tissues were used to prepare homogenates for the assessment of MDA, SOD, LDH, MMP-9, and IL-1β.

Additionally, the testis index (TI) was calculated according to the formula:

$$Testes\;index\;[\%]=\frac{testes\;weight\;[g]}{total\;body\;weight\;[g]}\times 100$$

**Figure 6:**
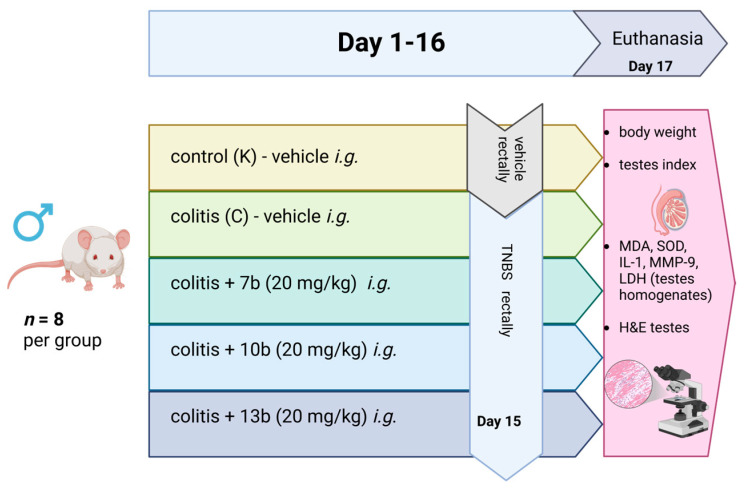
The general organization of the study. The vehicle used for intragastrical (*i.g.*) administration in groups K and C was 0.5% carboxymethylcellulose (CMC) solution; compounds 7b, 10b, and 13b were also suspended in 0.5% CMC solution in the equivalent volume. Group K received a normal saline solution rectally, and groups with induced colitis (C, 7b, 10b, and 13b) received an ethanol solution of 2,4,6-trinitrobenzenesulfonic acid (TNBS) in the same volume rectally. Created in BioRender. Nowak, B. (2025) https://BioRender.com/z6gkax3.

A general organization of the experiment is shown in Figure 6.

### 4.2. Tissue Homogenates Evaluation

One testis from each animal was homogenized on ice with phosphate buffer prepared according to the instructions from the powder (10× Bufor PBS—MIX, Syngen Biotech Sp. z o.o., Wrocław, Poland) and, thereafter, centrifuged at 5000 rpm for 15 min at 4 °C using the homogenizer Pro250 (Pro Scientific Inc., Oxford, CT, USA). Supernatants were collected, and a second homogenization at 4 °C was performed using the Ultrasonic processor UP100H (Hielscher Ultrasonics GmbH, Teltow, Germany). Supernatants for analysis were obtained after the second centrifugation at 12,000 rpm for 10 min. MDA, SOD, IL-1β, and MMP-9 were assayed in supernatants following the manufacturer’s instructions using Epoch ELISA Reader (BioTek Instruments, Winooski, VT, USA). SOD, as an enzyme, was expressed as ng/mg of protein. Total protein concentration and LDH activity in supernatants were measured in a certified laboratory using the Dimension RxL-Max (Siemens Healthineers Nederland B.V., Den Haag, The Netherlands). Total protein concentration was measured as mg/mL, and LDH activity was recalculated for U/mg of protein. The following ELISA kits were used in the study:

MDA—ELISA Kit for Malondialdehyde (MDA) (Cloud-Clone Corp., Wuhan, China).

SOD—Rat Superoxide Dismutase (SOD) ELISA (RCloud-Clone Corp., Wuhan, China).

IL-1β—Nori^®^ Rat IL-1 beta ELISA Kit (Genorise Scientific, Inc., Glen Mills, PA, USA).

MMP-9—Nori^®^ Rat MMP-9 ELISA Kit (Genorise Scientific, Inc., Glen Mills, PA, USA).

### 4.3. Histological Evaluation

After fixing the testes in 4% buffered formalin, they were prepared for histological assessment by embedding them in paraffin. Four-micrometer slices were stained with hematoxylin and eosin. A pathologist evaluated all samples blindly. An Olympus BX41 (Olympus Corporation, Tokyo, Japan) microscope with a Fujitsu computer system (Fijitsu, Tokyo, Japan) was used. The images of the representative samples were taken at 100× magnification.

### 4.4. Statistical Analysis

The impact of the compounds on the evaluated parameters was assessed using the Kruskal–Wallis ANOVA test, Dunn’s post hoc test, and Bonferroni correction. Hypotheses were positively verified if *p* < 0.05. TIBCO STATISTICA 13.3 PL (StatSoft, Kraków, Poland). Figure 1, Figure 2 and Figure 3 were created using GraphPad Prism version 10.4.1 (GraphPad Software, San Diego, CA, USA), and Figure 5 and Figure 6 were created using BioRender (Science Suite Inc. DBA BioRender, Toronto, ON, Canada). The experimental data in the figures are presented as means ± standard deviations (SD).

## 5. Conclusions

Despite some limitations of our study, based on the results obtained, we may conclude that the new 1,3,4-derivatives of pyrrolo[3,4-*d*]pyridazinone exert an insignificant harmful impact on testicular tissue. We revealed that compound 10b increased LDH activity, which requires further examination, while compound 13b increased the relative weight of the testes. Future studies solely designed to assess the toxicity of these compounds in the reproductive system, including semen morphology and motility, will be considered.

## Data Availability

The original contributions presented in the study are included in the article, further inquiries can be directed to the corresponding authors.
